# Visualization analysis of research hotspots and trends in traditional Chinese medicine external therapies for irritable bowel syndrome

**DOI:** 10.3389/fmed.2026.1954763

**Published:** 2026-09-23

**Authors:** Wang Rubing, Zhao Jinying, Kong Lingzu, Feng Xu, Yu Wenhui, Wang Ziheng, Feng Liqian, Wang Fuchun, Jiang Hailin

**Affiliations:** College of Acupuncture and Tuina, Changchun University of Chinese Medicine, Changchun, China

**Keywords:** citespace, external therapies in traditional Chinese medicine, irritable bowel syndrome, research hotspots, research trends, visual analysis

## Abstract

**Objective:**

To investigate the current status and future trends of research on external therapies in Traditional Chinese Medicine (TCM) for the treatment of irritable bowel syndrome, and to provide a reference for further research in this field.

**Methods:**

Relevant literature on the use of external therapies in Traditional Chinese Medicine for the treatment of irritable bowel syndrome was selected from the China National Knowledge Infrastructure (CNKI) database, the Web of Science database, and the PubMed database as the study subjects. CiteSpace was used to conduct a bibliometric analysis.

**Results:**

After manually screening duplicate literature using CiteSpace software, a total of 782 English-language articles and 963 Chinese-language articles were included. Analysis of the English-language articles revealed that the core author was Wu,Huan-Gan, and the institution with the highest output was Chengdu University of Traditional Chinese Medicine. Analysis of the Chinese articles revealed that the core author was Sun Jianhua, and the institution with the highest output was Shandong University of Traditional Chinese Medicine; the overall publication volume showed an upward trend. Keyword analysis indicated that research hotspots in this field include acupuncture, acupoint thread implantation, and symptoms associated with IBS, while research trends focus on the mechanisms underlying the treatment of IBS with external TCM therapies, principles for acupoint selection, and moxibustion therapy.

**Conclusion:**

The results of this study are consistent with the relevant literature on the treatment of irritable bowel syndrome (IBS) using external TCM therapies. By analyzing the research hotspots and trends in this field, this study highlights directions for further exploration and provides new directions and insights to guide clinical practice.

## 1 Introduction

Irritable bowel syndrome (IBS) is a functional bowel disorder (FBD) characterized by recurrent abdominal pain, the severity of which is related to bowel movements. Patients typically exhibit irregular bowel habits, such as constipation, diarrhea, or a combination of both, often accompanied by abdominal bloating or a sensation of fullness (1, 2). Epidemiological studies indicate that approximately 11.2% of adults worldwide suffer from IBS, and the prevalence is increasing year by year (3). Relevant research shows that this condition is characterized by a prolonged treatment duration and a high recurrence rate, which places economic and psychological strain on patients while simultaneously reducing their quality of life (4, 5). Statistical analyses confirm that IBS patients often exhibit symptoms of mental disorders such as depression and anxiety (6, 7). Currently, the primary medications used to treat IBS include antibiotics, antidepressants, and antispasmodic serotonin 3-receptor antagonists; however, these drugs have significant side effects. For example, antibiotics can lead to drug resistance, while antidepressants may cause drowsiness and even exacerbate constipation symptoms (8–11).

Traditional Chinese Medicine (TCM) external therapies represent a time-honored and unique system of traditional Chinese treatment. These therapies include acupuncture, electroacupuncture, moxibustion, tuina massage, auricular therapy, and acupoint plaster application. They are characterized by ease of administration, high safety, and high patient acceptance (12). TCM external therapies offer the following advantages in treating IBS: First, they can reduce adverse reactions and improve patient compliance (13). Second, they can simultaneously alleviate multiple symptoms in IBS patients, thereby reducing their suffering (14). Additionally, these therapies can ease the financial burden on IBS patients and improve their quality of life.

Citation metrics provide statistical analysis of popular subfields within a scientific discipline (15, 16). This method primarily involves the quantitative evaluation of academic literature, including the counting of citations, tracking publication trends, mapping co-author networks, and identifying emerging topics, which can be used to uncover the potential of a research field (17, 18). The literature on the treatment of IBS using TCM external therapies is extensive and complex; to date, no researcher has conducted a comprehensive citation metrics analysis of this field. This paper utilizes CiteSpace software to conduct a visual analysis of the relevant literature on the treatment of IBS using external TCM therapies.

CiteSpace is visualization software developed by Professor Chen Chaomei's team on the Java platform. The software is used to explore the evolution of scientific fields, ranging from single disciplines to multiple interrelated scientific frontiers (19). Visualization analysis is an analytical method that comprehensively and intuitively presents research data in graphical form. It is widely applied across various fields (20, 21). This study aims to enable researchers to quickly grasp the foundational knowledge system and the current state of research on the treatment of IBS using external methods in traditional Chinese medicine through literature analysis, while also providing guidance for future research.

## 2 Materials and methods

### 2.1 Literature sources

Computerized database searches were conducted in CNKI (China National Knowledge Infrastructure), PubMed, and Web of Science (WOS). Data on the treatment of irritable bowel syndrome (IBS) using external TC M therapies published between January 1990 and January 2026 were collected. Detailed search terms and strategies are shown in Table 1.

**Table 1:** Search strategies.

Database	Search term
PubMed	((((((((((((((((((((((((((((((((irritable bowel syndrome[Title/Abstract]) OR (patients with irritable bowel syndrome[Title/Abstract])) OR (IBS[Title/Abstract])) OR (irritable bowel syndrom[Title/Abstract])) OR (irritable bowel disease[Title/Abstract])) OR (irritable bowel syndromes[Title/Abstract])) OR (irritable bowl syndrome[Title/Abstract])) OR (irritable bowel syndrome with diarrhea[Title/Abstract])) OR (irritable colon syndrome[Title/Abstract])) OR (irritable bower syndrome[Title/Abstract])) OR (predominant irritable bowel syndrome[Title/Abstract])) OR (irritable bowel[Title/Abstract])) OR (epidemiology of irritable bowel syndrome[Title/Abstract])) OR (iritable bowel syndrome[Title/Abstract])) OR (diarrhea-type irritable bowel syndrome[Title/Abstract])) OR (intestinal irritable syndrome[Title/Abstract])) OR (intestinal irritability syndrome[Title/Abstract])) OR (intestinal stress syndrome[Title/Abstract])) OR (constipation type of irritable bowel syndrome[Title/Abstract])) OR (predominant pattern irritable bowel syndrome[Title/Abstract])) OR (irritable bowels syndrome[Title/Abstract])) OR (bowel irritability syndrome[Title/Abstract])) OR (intestine irritability syndrome[Title/Abstract])) OR (irritable bwel syndrome[Title/Abstract])) OR (irritable bowel syndrone[Title/Abstract])) OR (irritable syndrome[Title/Abstract])) OR (bowel syndrome[Title/Abstract])) OR (intestine irritable syndrome[Title/Abstract])) OR (irritable bowed syndrome[Title/Abstract])) OR (syndrome of intestines surge[Title/Abstract])) OR (diarrhea-dominant irritable bowel syndrome[Title/Abstract])) OR (irritable bowel syndome[Title/Abstract])) OR (diarrhea-predominant irritable bowel syndrome[Title/Abstract]) AND ((((((((((((((((((acupuncture[Title/Abstract]) OR (electric acupuncture[Title/Abstract])) OR (silver needle[Title/Abstract])) OR (tuina[Title/Abstract])) OR (moxibustion[Title/Abstract])) OR (moxa[Title/Abstract])) OR (cupping therapy[Title/Abstract])) OR (cupping[Title/Abstract])) OR (gua sha[Title/Abstract])) OR (rub sha[Title/Abstract])) OR (scrapping therapy[Title/Abstract])) OR (auricular-plaster[Title/Abstract])) OR (auricular point sticking[Title/Abstract])) OR (auricular point pressing[Title/Abstract])) OR (ear acupoint[Title/Abstract])) OR (acupoint application[Title/Abstract])) OR (acupressure points[Title/Abstract])) OR (acupuncture point applying[Title/Abstract])) OR (external therapies in traditional Chinese medicine[Title/Abstract])
Wob of science	#1:TS = (irritable bowel syndrome)
	#2:TS = (patients with irritable bowel syndrome)
	#3:TS = (IBS)
	#4:TS = (irritable bowel syndrom)
	#5:TS = (irritable bowel disease)
	#6:TS = (irritable bowel syndromes)
	#7:TS = (irritable bowl syndrome)
	#8:TS = (irritable bowel syndrome with diarrhea)
	#9:TS = (irritable colon syndrome)
	#10:TS = (irritable bower syndrome)
	#11:TS = (predominant irritable bowel syndrome)
	#12:TS = (irritable bowel)
	#13:TS = (epidemiology of irritable bowel syndrome)
	#14:TS = (iritable bowel syndrome)
	#15:TS = (diarrhea-type irritable bowel syndrome)
	#16:TS = (intestinal irritable syndrome)
	#17:TS = (intestinal irritability syndrome)
	#18:TS = (intestinal stress syndrome)
	#19:TS = (constipation type of irritable bowel syndrome)
	#20:TS = (predominant pattern irritable bowel syndrome)
	#21:TS = (irritable bowels syndrome)
	#22:TS = (bowel irritability syndrome)
	#23:TS = (intestine irritability syndrome)
	#24:TS = (irritable bwel syndrome)
	#25:TS = (irritable bowel syndrone)
	#26:TS = (irritable syndrome)
	#27:TS = (bowel syndrome)
	#28:TS = (intestine irritable syndrome)
	#29:TS = (irritable bowed syndrome)
	#30:TS = (syndrome of intestines surge)
	#31:TS = (diarrhea-dominant irritable bowel syndrome)
	#32:TS = (irritable bowel syndome)
	#33:TS = (diarrhea-predominant irritable bowel syndrome)
	#34:#1 OR #2 OR #3 OR #4 OR #5 OR #6 OR #7 OR #8 OR #9 OR #10 OR #11 OR #12 OR #13 OR #14 OR #15 OR #16 OR #17 OR #18 OR #19 OR #20 OR #21 OR #22 OR #23 OR #24 OR #25 OR #26 OR #27 OR #28 OR #29 OR #30 OR #31 OR #32 OR #33
	#35:TS = (acupuncture)
	#36:TS = (electric acupuncture)
	#37:TS = (silver needle)
	#38:TS = (tuina)
	#39:TS = (moxibustion)
	#40:TS = (moxa)
	#41:TS = (cupping therapy)
	#42:TS = (cupping)
	#43:TS = (gua sha)
	#44:TS = (rub sha)
	#45:TS = (scrapping therapy)
	#46:TS = (auricular-plaster)
	#47:TS = (auricular point sticking)
	#48:TS = (auricular point pressing)
	#49:TS = (ear acupoint)
	#50:TS = (acupoint application)
	#51:TS = (acupressure points)
	#52:TS = (acupuncture point applying)
	#53:TS = (external therapies in traditional Chinese medicine)
	#54:#35 OR #36 OR #37 OR #38 OR #39 OR #40 OR #41 OR #42 OR #43 OR #44 OR #45 OR #46 OR #47 OR #48 OR #49 OR #50 OR #51 OR #52 OR #53
	#55:#34 AND #54
CNKI	#1:SU % = “肠易激综合征” OR SU % = “腹泻型肠易激综合征” OR SU % = “便秘型肠易激综合征” OR SU % = “混合型肠易激综合征”
	#2: SU % = “针刺” OR SU % = “针灸” OR SU % = “推拿” OR SU % = “手法” OR SU % = “按摩” OR SU % = “埋线” OR SU % = “火罐” OR SU % = “刮痧” OR SU% = “耳穴压豆” OR SU % = “穴位贴敷” OR SU % = “中医外治法”
	#3:#1 AND #2

### 2.2 Article selection criteria

#### 2.2.1 Inclusion criteria

The inclusion criteria were as follows: (i) articles related to TCM external therapies for irritable bowel syndrome; (ii) journal articles; and (iii) articles containing essential information such as publication date, authors, and keywords.

#### 2.2.2 Exclusion criteria

The exclusion criteria were as follows: (i) duplicated publications; (ii) literature whose title was unrelated to the treatment of irritable bowel syndrome using TCM external therapies; and (iii) conference papers, patents, news articles, advertisements, and popular science articles.

### 2.3 Data analysis

Data from the CNKI database was exported in “RefWorks” format. The “RefWorks” format database was imported into the built-in file format converter of the CiteSpace software and converted to the `download_.TXT` format for use. Data from the WOS and PubMed databases was exported in WOS plain text format. The search and organization functions of the CiteSpace software (and) were used to search for and organize articles from both databases. Import the data into CiteSpace and set the following parameters: time partition 1990–2026; time slice (year), “1”; and node types: author, institution, and keyword. This enables knowledge graph analysis of article authors, institutions, countries, keywords, and other aspects.

## 3 Results

### 3.1 Visualization of annual publication volumes

The annual number of publications is a key indicator for summarizing past research and predicting future research trends in the field. Using CiteSpace, duplicates were removed, and the final dataset of 782 English-language articles and 963 Chinese-language articles was analyzed (Figure 1).

**Figure 1:**
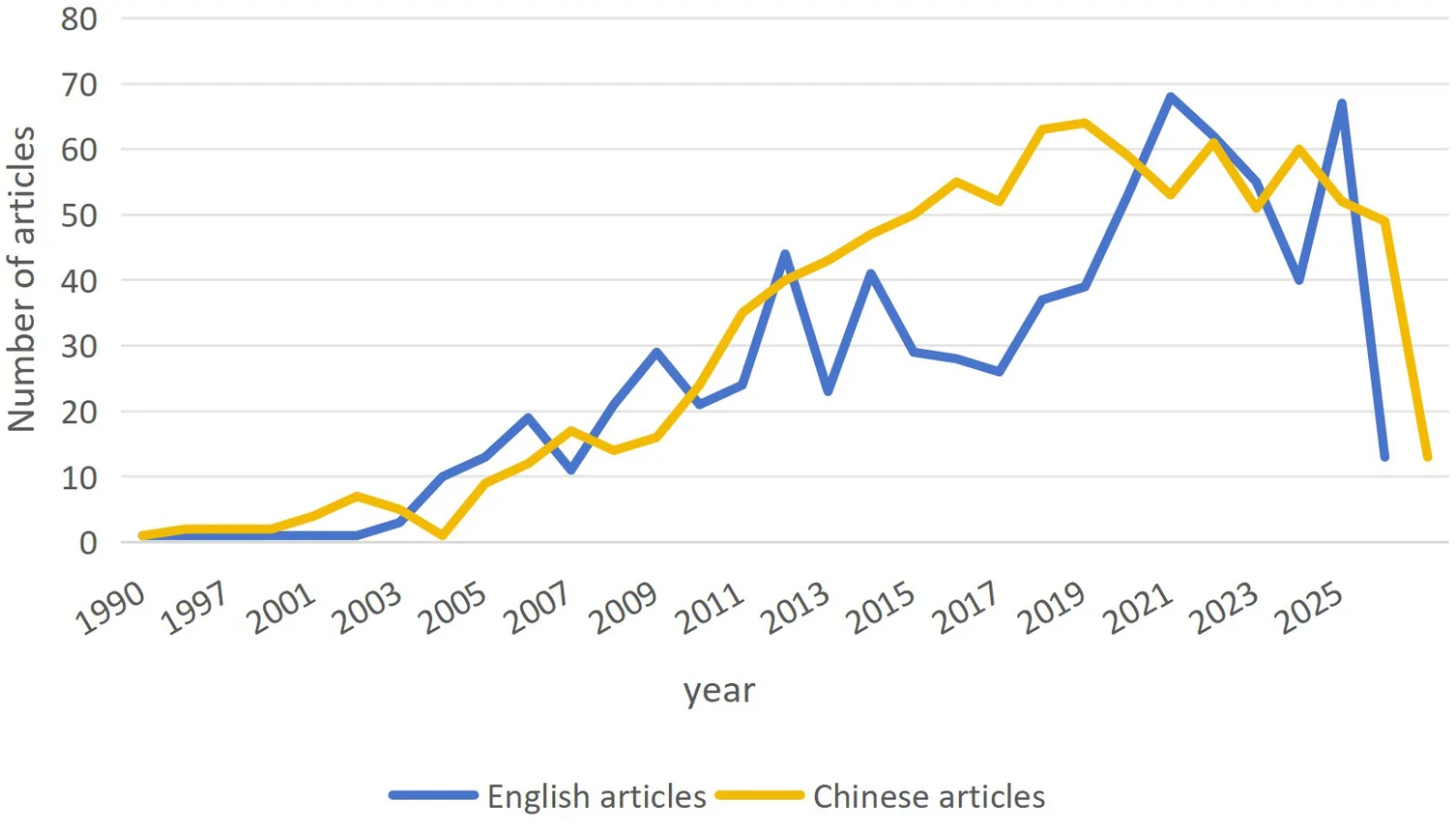
Annual articles on the combination of acupuncture and traditional Chinese medicine therapy.

### 3.2 Visual mapping of author publication output

Using CiteSpace software to analyze the authors of Chinese and English articles on the treatment of IBS with traditional Chinese external therapies allows us to identify core researchers in this field and understand the collaboration patterns among authors. In the visualization, the size of network nodes reflects an author's publication output—that is, the more articles an author has published, the larger the node—while the thickness of the connecting lines reflects the frequency of collaboration between authors. Additionally, the visualization highlights influential research groups and potential co-authors, thereby helping researchers establish collaborative networks.

Using CiteSpace software to analyze the authors of Chinese and English articles, we generated author collaboration network diagrams (Figures 2A,B) and listed the top 10 authors by number of published articles based on these diagrams (Table 2). In the collaboration network diagram for English-language articles (Figure 2A), there are 90 nodes, 106 links, and a network density of 0.0265; in the collaboration network diagram for Chinese-language articles (Figure 2B), there are 111 nodes, 121 links, and a network density of 0.0198.

**Figure 2:**
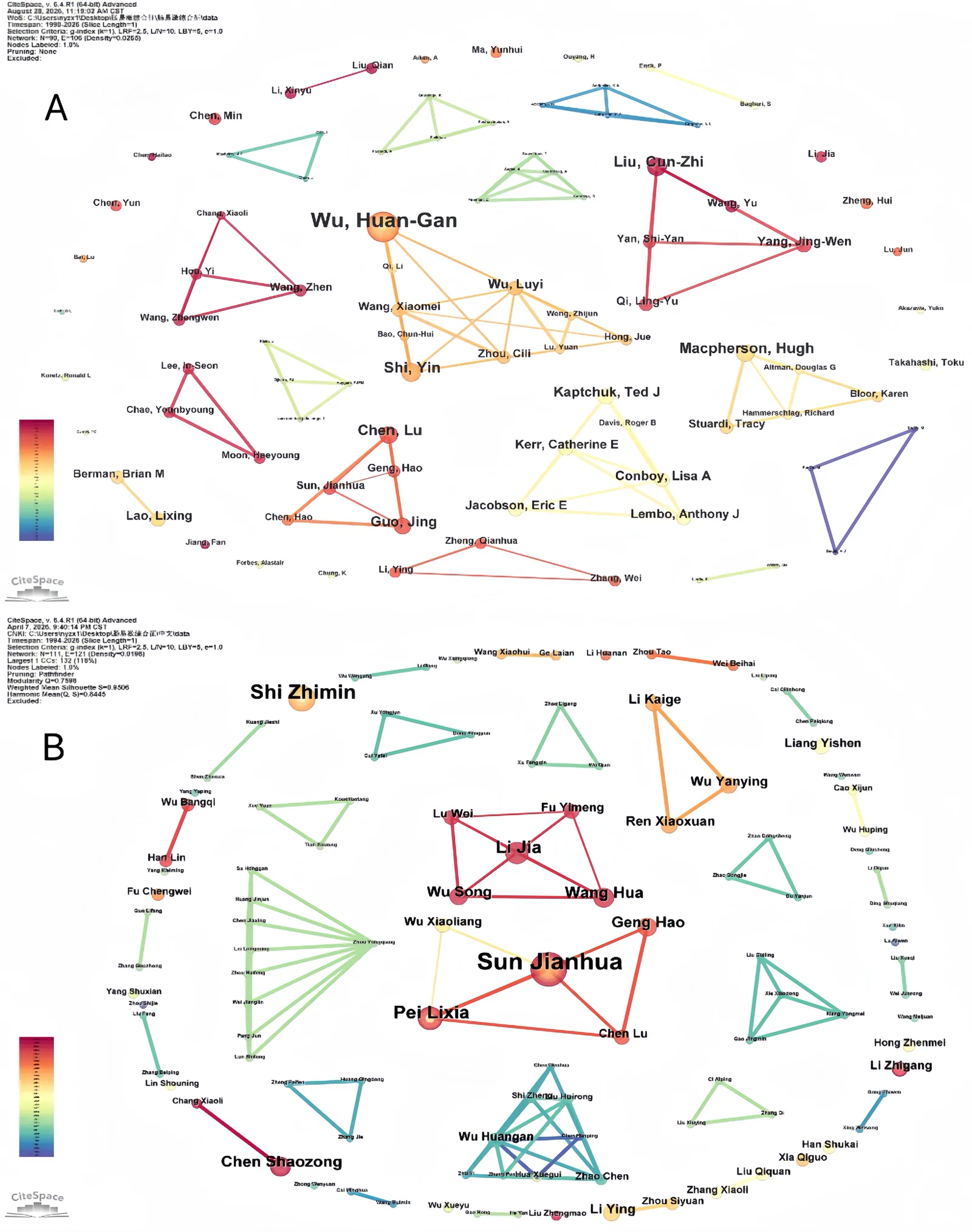
**(A)** Network map of authors of english literature; **(B)** network map of authors of Chinese literature.

**Table 2:** The top 10 authors publishing articles.

No.	English literature		Chinese literature	
	Author	Frequency	Author	Frequency
1	Wu, Huan-Gan	37	Sun Jianhua	28
2	Shi, Yin	13	Shi Zhimin	15
3	Liu, Cun-Zhi	13	Pei Lixia	12
4	Chen, Lu	11	Li Jia	10
5	Macpherson, Hugh	11	Chen Shaozong	8
6	Guo, Jing	10	Wang Hua	8
7	Kaptchuk, Ted J	9	Geng Hao	7
8	Conboy, Lisa A	8	Wu Song	6
9	Wu, Luyi	8	Li Kaige	5
10	Jacobson, Eric E	7	Li Ying	5

### 3.3 Visual mapping of institutional publication output

Institutional analysis using CiteSpace software identifies the core research institutions in this field and reveals the nature of collaboration among them. In the figure, the size of each node reflects an institution's publication output—the larger the node, the greater the output—while the lines represent collaborative relationships between institutions; the thicker the line, the more frequent the collaboration. This figure provides a visual representation of influential collaborating institutions.

Using CiteSpace software to analyze the institutions associated with Chinese and English articles, we generated institutional collaboration network diagrams (Figures 3A,B) and listed the top 10 authors by number of published articles based on these diagrams (Table 3). In the institutional collaboration network diagram for English-language articles (Figure 3A), there are 62 network nodes, 36 connections, and a network density of 0.019; in the institutional collaboration network diagram for Chinese-language articles (Figure 3B), there are 55 network nodes, 4 connections, and a network density of 0.0027.

**Figure 3:**
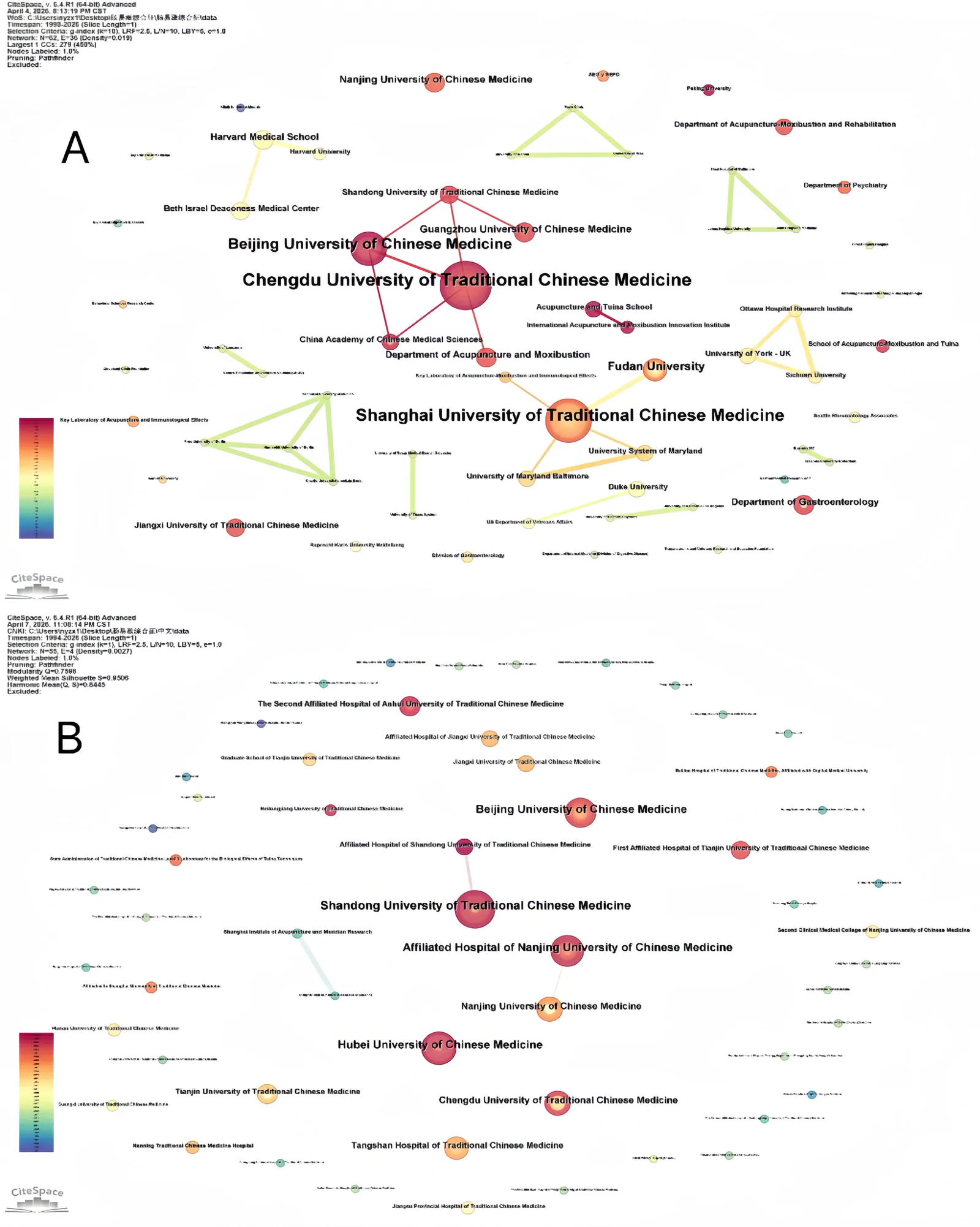
**(A)** Network map of institutions of english literature; **(B)** network map of institutions of Chinese literature.

**Table 3:** The top 10 institutions publishing articles.

No.	English literature		Chinese literature	
	Author	Frequency	Author	Frequency
1	Chengdu University of Traditional Chinese Medicine	41	Shandong University of Traditional Chinese Medicine	23
2	Shanghai University of Traditional Chinese Medicine	36	Hubei University of Chinese Medicine	20
3	Beijing University of Chinese Medicine	27	Affiliated Hospital of Nanjing University of Chinese Medicine	19
4	Fudan University	10	Beijing University of Chinese Medicine	18
5	Guangzhou University of Chinese Medicine	7	Nanjing University of Chinese Medicine	11
6	Nanjing University of Chinese Medicine	7	Chengdu University of Traditional Chinese Medicine	10
7	Department of Acupuncture and Moxibustion	6	Tangshan Hospital of Traditional Chinese Medicine	9
8	Harvard Medical School	6	Tianjin University of Traditional Chinese Medicine	6
9	Department of Gastroenterology	6	The Second Affiliated Hospital of Anhui University of Traditional Chinese Medicine	6
10	Jiangxi University of Traditional Chinese Medicine	5	Affiliated Hospital of Shandong University of Traditional Chinese Medicine	5

### 3.4 Visual mapping of keyword networks

#### 3.4.1 Visual mapping of keyword co-occurrence

Based on the analysis conducted using CiteSpace software, co-occurrence maps of Chinese and English keywords were generated (Figures 4A,B), and the keywords were ranked by frequency; node size represents the frequency of keyword occurrence (Table 4). In the collaboration network graph for English-language articles (Figure 4A), there are 294 nodes, 592 edges, and a network density of 0.0137; in the collaboration network graph for Chinese-language articles (Figure 4B), there are 139 nodes, 180 edges, and a network density of 0.0188.

**Figure 4:**
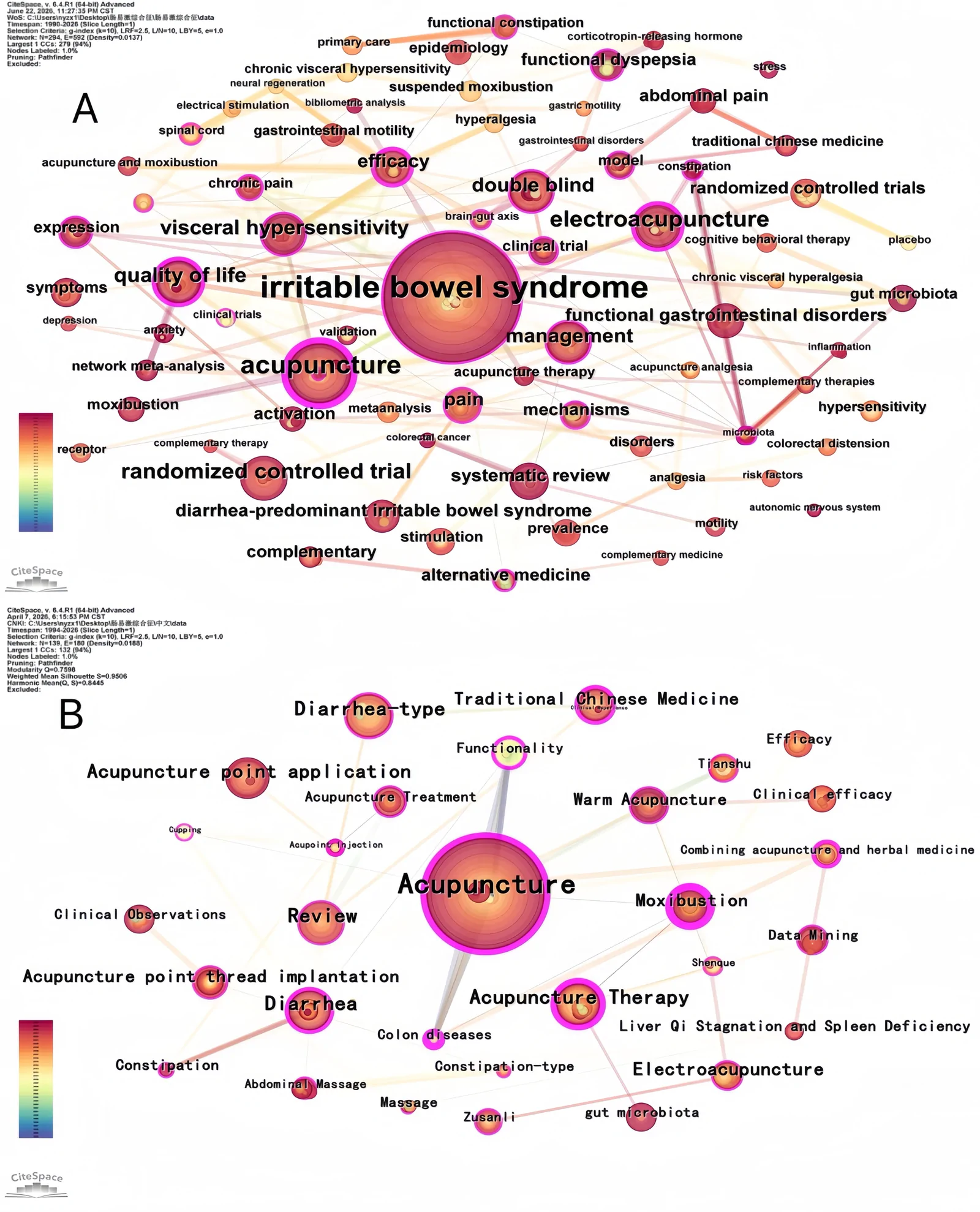
**(A)** Keyword co-occurrence mapping in english literature; **(B)** keyword co-occurrence mapping in Chinese literature.

**Table 4:** The top 10 frequency-ranking keywords.

No.	English literature		Chinese literature	
	Keyword	Frequency	Keyword	Frequency
1	irritable bowel syndrome	378	Acupuncture	370
2	acupuncture	118	Diarrhea	71
3	electroacupuncture	67	Review	70
4	randomized controlled trial	53	Diarrhea-type	69
5	quality of life	48	Acupuncture Therapy	61
6	visceral hypersensitivity	43	Acupuncture point application	52
7	management	35	Electroacupuncture	41
8	double blind	34	Traditional Chinese Medicine	40
9	efficacy	28	Moxibustion	35
10	pain	28	Acupuncture point thread implantation	34

#### 3.4.2 Visual mapping of keyword clusters

CiteSpace software was used to extract keywords from the articles for clustering. The Q-value is the clustering module value, a metric that measures clustering quality and reflects the compactness of the clustering results. A Q-value >0.3 indicates a relatively distinct clustering structure. The S-value represents the average silhouette of the clusters and is used to evaluate the similarity and dissimilarity of nodes in the clustering results. An S-value >0.5 indicates that the clustering results are reasonable; an S-value >0.7 suggests that the clustering results are highly reliable. For the English articles, the values for the keyword clustering diagram (Figure 5A) are: *Q* = 0.7979, *S* = 0.9213. For the Chinese articles, the values for the keyword clustering diagram (Figure 5B) are: *Q* = 0.7598, *S* = 0.9506. The data indicate that the clustering structure in these diagrams is significant and that the clustering results are highly reliable.

**Figure 5:**
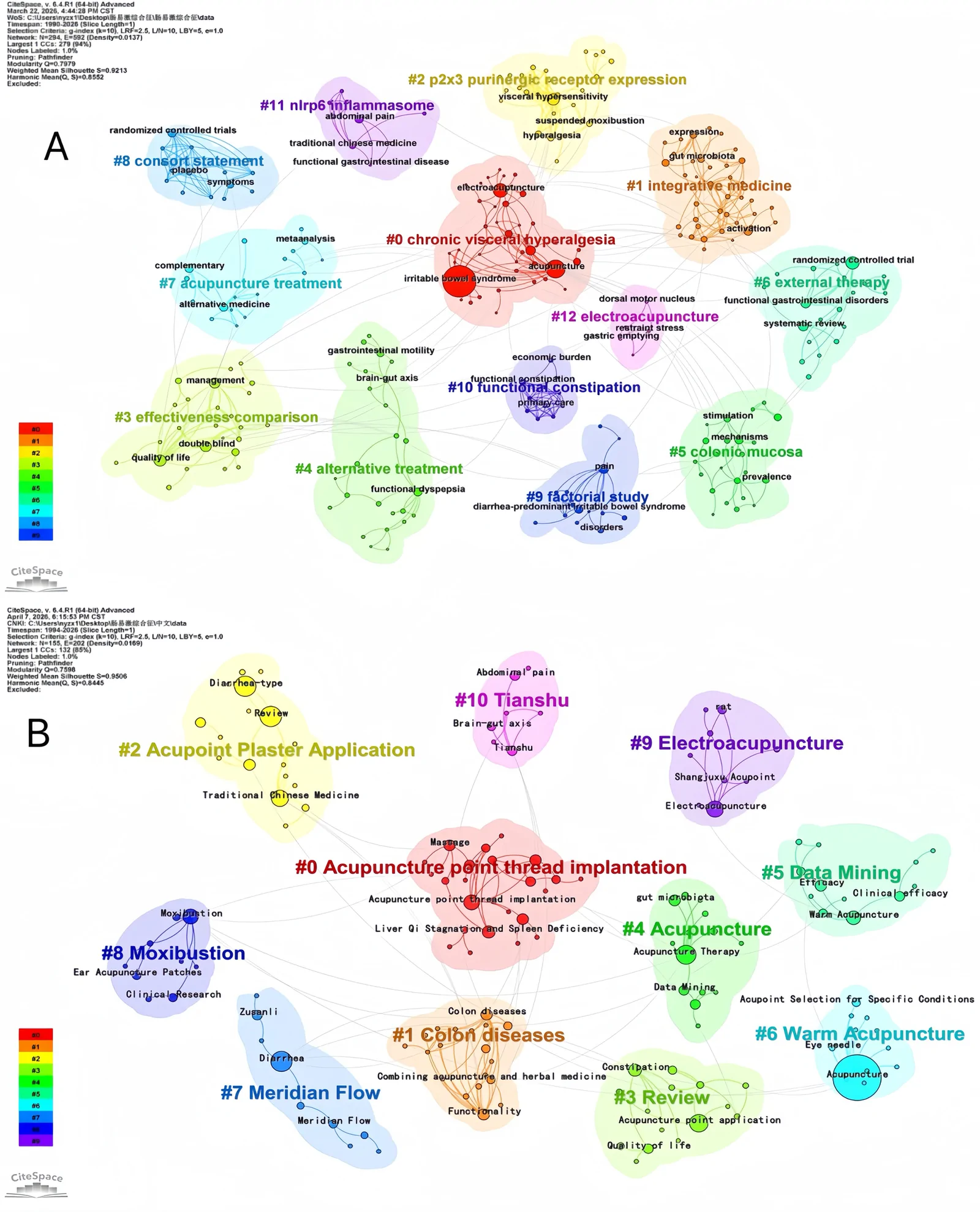
**(A)** Keyword clustering mapping in english literature; **(B)** keyword clustering mapping in Chinese literature.

The keyword timeline visualization maps intuitively display the changes in keywords within each cluster over time. The horizontal axis represents time, and the vertical axis displays cluster names. CiteSpace software was used to cluster keywords from Chinese and English articles and generate timeline plots (Figures 6A,B).

**Figure 6:**
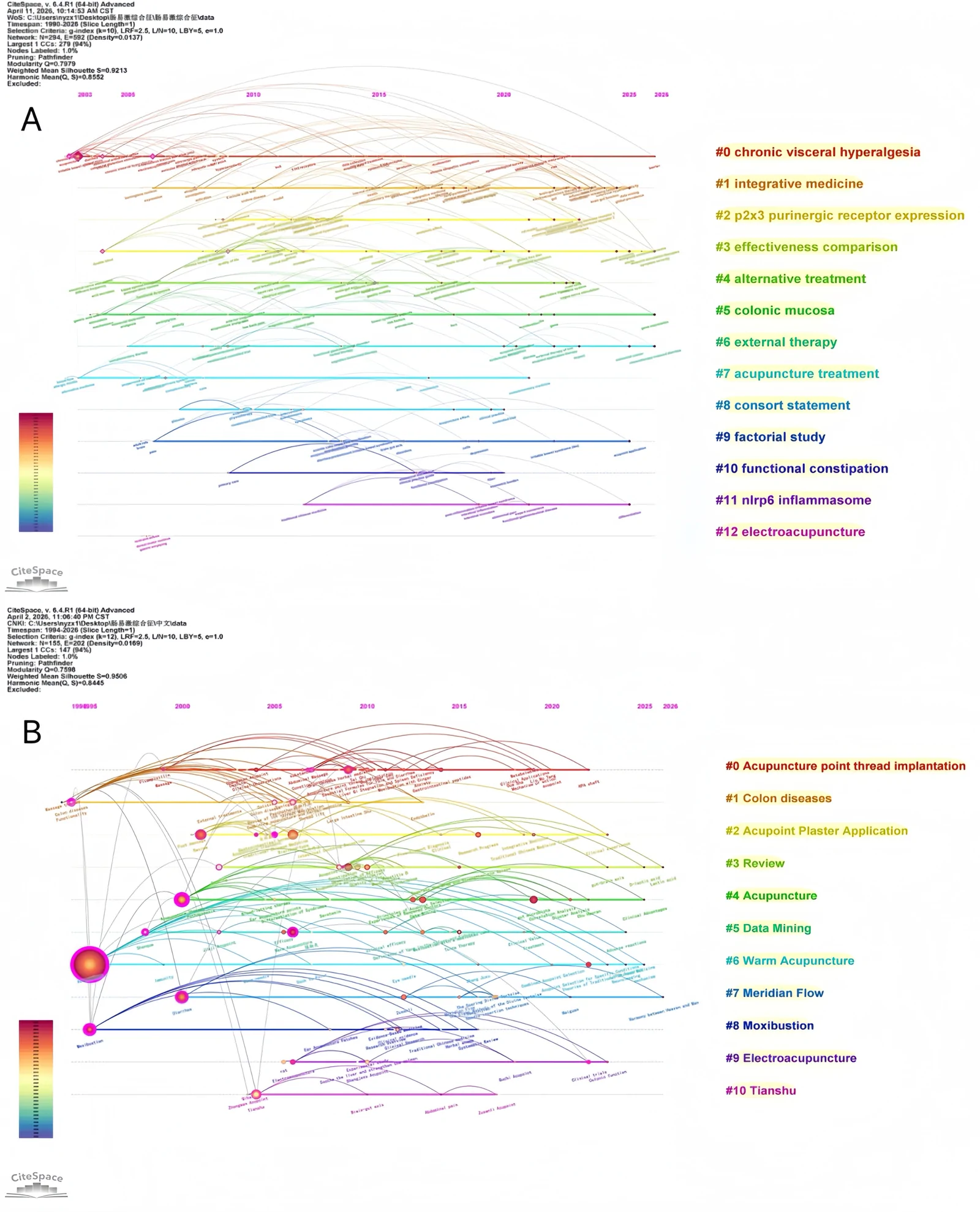
**(A)** Keyword clustering timeline mapping in english literature; **(B)** keyword clustering timeline mapping in Chinese literature.

#### 3.4.3 Visual mapping of keyword burst charts

Keyword bursts refer to keywords that are repeatedly mentioned and discussed during a specific period; these keywords help researchers identify research hotspots in a given field during a particular time and predict future research trends. CiteSpace software was used to perform burst analysis on the keywords of Chinese and English articles, generating keyword burst plots (Figures 7A,B).

**Figure 7:**
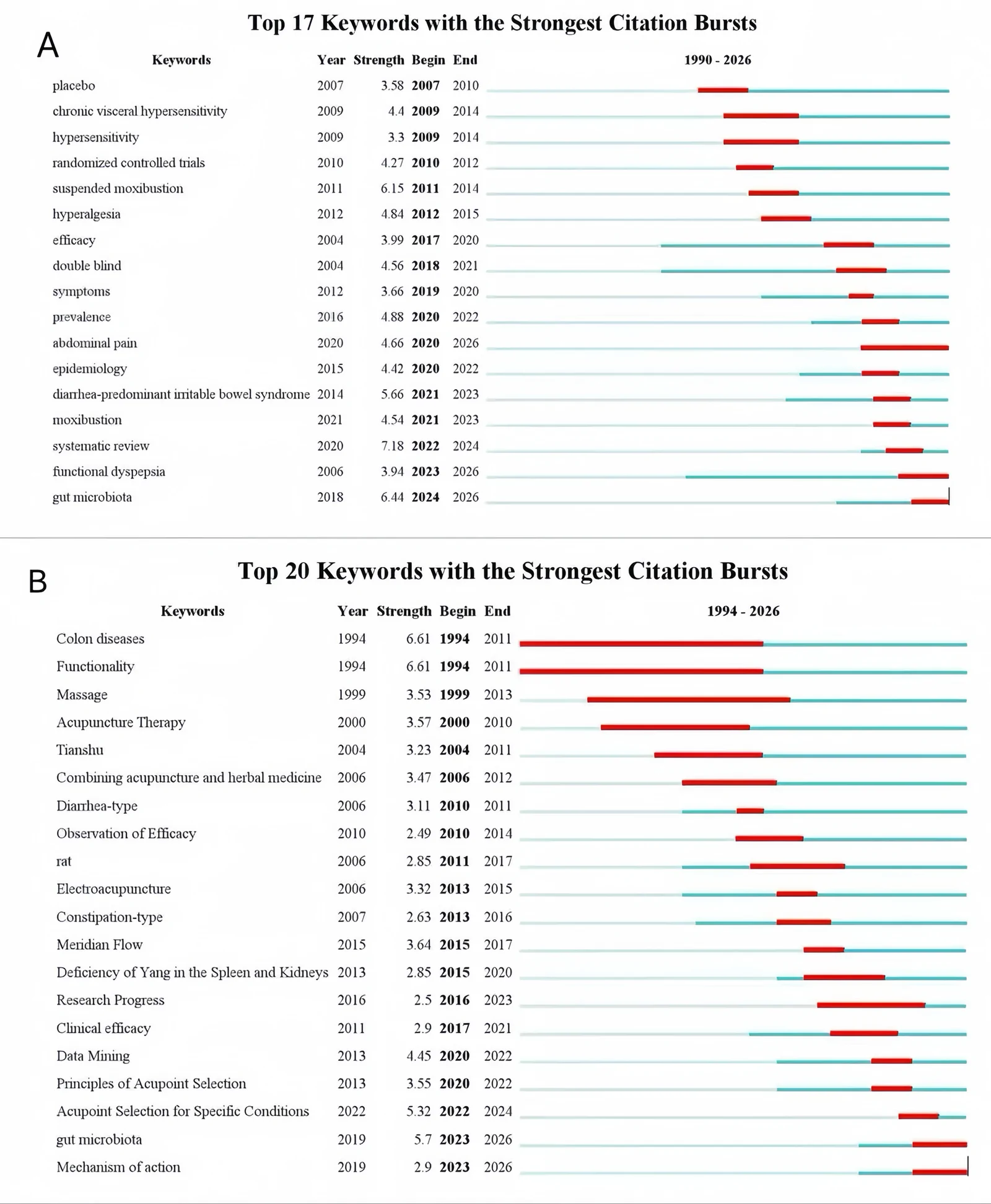
**(A)** Keyword bursting mapping in english literature; **(B)** keyword bursting mapping in Chinese literature.

## 4 Discussion

### 4.1 Research overview

#### 4.1.1 Analysis of annual publication volume

An analysis of relevant English-language articles in this field revealed that the number of published papers grew steadily from 2005 to 2026. From 2017 to 2025, the number of published papers grew rapidly, peaking in 2022.The discussion primarily focuses on electroacupuncture and acupuncture. Analysis of relevant Chinese articles in this field shows that from 2005 to 2026, the number of Chinese publications grew steadily, with a rapid increase in the number of papers published from 2009 to 2026, peaking in 2019. The discussion centers on acupuncture therapy, diarrhea symptoms, and the application of acupuncture points. The total number of published papers on the treatment of IBS using external TCM therapies shows an upward trend, indicating that this field is attracting increasing attention.

#### 4.1.2 Analysis of authors' publication output

An analysis of publication output by authors of English-language articles revealed that the top five most prolific authors were Wu,Huan-Gan; Shi, Yin; Liu, Cun-Zhi; Chen, Lu and Macpherson, Hugh. Among them, Kaptchuk, Ted J. and Wu,Huan-Gan collaborated most closely with other authors. An analysis of publication output by authors of Chinese-language articles revealed that the top five authors were Sun Jianhua, Shi Zhimin, Pei Lixia, Li Jia, and Chen Shaozong. Among them, Zhou Yongqiang and Wu Huanguan collaborated most closely with other authors.

A core author is defined as a researcher in a given field who produces high-quality publications and possesses strong research capabilities and influence. According to Price's Law, the formula for identifying core authors is: M_p_ = 0.749√N_pmax_. Here, M_(p)_ represents the minimum number of articles published by a core author, and N_pmax_represents the number of articles published by the author with the highest publication output.

Among authors publishing in English, Wu,Huan-Gan have published the most articles, _with_ N_pmax_ = 37. According to the formula, M_p_ ≈ 4.6 articles. Thus, authors who have published five or more articles in this field are considered core authors. There are a total of 25 core authors, who have published a combined total of 215 articles. The output of core authors accounts for 27.4% of the total output of all authors, a value that is <50%. Among authors who published Chinese-language articles, Sun Jianhua published the most articles, with N_pmax_ = 28. According to the formula, M_p_ ≈ 3.96 articles. Thus, authors who published four or more articles in this field are considered core authors. There are 17 core authors who have published a total of 127 articles. The number of articles published by core authors accounts for 13.1% of the total number of articles published by all authors, a figure that is less than 50%. This study summarizes and analyzes the publication output of authors of Chinese and English articles in this field and concludes that a stable group of core authors has not yet emerged. Further research should be conducted to improve research quality and enhance collaboration among authors in order to promote the further development of this field.

### 4.1.3 Analysis of institutional publication output

An analysis of the number of papers published by institutions in English-language articles revealed that the top five institutions by publication volume were Chengdu University of Traditional Chinese Medicine, Shanghai University of Traditional Chinese Medicine, Beijing University of Chinese Medicine, Fudan University, and Guangzhou University of Traditional Chinese Medicine. The top ten institutions accounted for a total of 151 papers, representing only 19.3% of the total number of articles. Additionally, Chengdu University of Traditional Chinese Medicine and Shanghai University of Traditional Chinese Medicine engaged in relatively extensive collaboration with other institutions.

An analysis of the number of Chinese-language articles published by institutions revealed that the top five institutions by publication volume were Shandong University of Traditional Chinese Medicine, Hebei University of Traditional Chinese Medicine, the Affiliated Hospital of Nanjing University of Traditional Chinese Medicine, Beijing University of Chinese Medicine, and Nanjing University of Traditional Chinese Medicine. The top ten institutions accounted for a total of 151 publications, representing only 19.3% of the total number of articles, indicating that the distribution of publishing institutions is relatively dispersed. The top ten institutions accounted for a total of 127 publications, representing only 13.1% of the total number of articles. This suggests that the publishing institutions for Chinese-language articles are relatively dispersed, and there are disparities in research capabilities among various research institutions.

### 4.2 Keyword network analysis

Keywords encapsulate the main content of an article. This study analyzed the keywords in relevant literature to summarize research hotspots in the field and predict research trends.

Analysis using CiteSpace software revealed that the top five high-frequency keywords in English articles were “irritable bowel syndrome,” “acupuncture,” “electroacupuncture,” “randomized controlled trial,” and “quality of life.” The top five high-frequency keywords in Chinese articles were “acupuncture,” “diarrhea,” “review,” “diarrhea-type,” and “acupuncture therapy.”

Analysis of the visual keyword clustering maps for both English and Chinese articles revealed that, in English articles, #0chronic visceral hyperalgesia and #10functional constipation are relatively common clinical manifestations of IBS. #1integrative medicine, #4alternative treatment, #6external therapy, #7acupuncture treatment, and #12electroacupuncture are the treatment methods used in clinical practice. #2p2 × 3 purinergic receptor expression, #5colonic mucosa, and #11NLRP6 inflammasome represent research on the mechanisms underlying the treatment of IBS using external therapies in traditional Chinese medicine. #3effectiveness comparison, #8CONSORT statement, and #9factorial study represent different methods used in animal experiments and clinical trials.

In the Chinese articles, #1 Colon Diseases, #0 Acupoint Thread Implantation, #2 Acupoint Plaster Application, #4 Acupuncture, #6 Warm Acupuncture, #8 Moxibustion, and #9 Electroacupuncture are the primary external TCM treatment methods for IBS used in clinical practice.#3Review and #5Data Mining are methods for analyzing relevant data. #7Meridian Flow and #10Tianshu represent the primary theoretical foundations and acupoint selection for TCM external therapies in treating IBS.

Based on a visual analysis of the keyword emergence map:In English-language articles, no burst keywords appeared in the early phase, suggesting that research on the use of traditional Chinese medicine external therapies for treating IBS was relatively scattered during that period. Research in the middle phase primarily focused on “placebo,” “chronic visceral hypersensitivity,” “hypersensitivity,” “randomized controlled trials,” “suspended moxibustion,” “hyperalgesia,” “efficacy,” “double-blind,” “symptoms,” and “prevalence”;late-stage research primarily focused on “abdominal pain,” “epidemiology,” “diarrhea-predominant irritable bowel syndrome,” “moxibustion,” “systematic review,” “functional dyspepsia,” and “gut microbiota.”In Chinese literature, early studies primarily focused on “colon diseases,” “functionality,” “massage,” “acupuncture therapy,” “Tianshu,” “combining acupuncture and herbal medicine,” and “diarrhea-type”;Mid-period studies primarily focused on “Observation of Efficacy,” “rat,” “electroacupuncture,” “constipation-type,” “meridian flow,” and “deficiency of Yang in the spleen and kidneys”;late-stage studies primarily focused on “Research Progress,” “Clinical Efficacy,” “Data Mining,” “Principles of Acupoint Selection,” “Acupoint Selection for Specific Conditions,” “gut microbiota,” and “Mechanism of Action.” Analysis of the keyword emergence map reveals that research trends in TCM treatment of IBS are primarily concentrated on mechanism studies, principles of acupoint selection, and moxibustion.

### 4.3 Hot topics

In recent years, research on irritable bowel syndrome (IBS) has primarily focused on acupuncture, acupoint thread implantation, and the accompanying symptoms of IBS.

#### 4.3.1 Acupuncture

Acupuncture is a Traditional Chinese Medicine method with a 5,000-year history that can effectively alleviate the clinical symptoms of irritable bowel syndrome (IBS) (22, 23). Relevant clinical studies show that acupuncture can relieve abdominal pain associated with IBS by reducing miR-148a-3p levels, and this therapy is associated with fewer side effects (24, 25). Currently, the Delphi expert consensus clearly indicates that acupuncture can effectively alleviate the clinical symptoms of mild and moderate IBS (26–28). It is worth noting that IBS is a chronic gastrointestinal disorder, and because patients with IBS experience long-term abdominal pain, approximately 30% to 50% of patients develop depression in the later stages of the disease (29, 30). Multiple animal studies have found that acupuncture can alleviate abdominal pain symptoms in patients with IBD by modulating the nervous system, and that this therapy can also alleviate depressive symptoms in rats with IBS by modulating both the nervous and immune systems (31, 32).

Relevant studies have shown that electroacupuncture and warm acupuncture are effective treatments for IBD (33–35). Electroacupuncture is a therapeutic method in which a pulse current is delivered via an electroacupuncture device after achieving “qi” through fine-needle acupuncture; this therapy transmits electrical current through the fine needles to specific areas of the body to treat diseases. Relevant studies have confirmed that electroacupuncture can alleviate abdominal pain symptoms in IBS patients while improving their quality of life (36). Multiple animal studies have confirmed that electroacupuncture can treat IBS by repairing the intestinal mucosa and inhibiting the proliferation and activation of mucosal mast cells (37–40). A recent objective transcriptomic study has confirmed that electroacupuncture can alleviate symptoms of intestinal mucosal damage in IBS patients through lncRNA-miRNA-mRNA regulation (41); this experiment provides new perspectives and insights for future research into the mechanisms of electroacupuncture in treating IBS.Analysis of relevant data confirms that the optimal duration of electroacupuncture therapy for treating IBS is 30 min. Notably, different pulse combinations can alleviate symptoms of different IBS subtypes: expansion waves effectively relieve abdominal pain in patients with IBS-D, while intermittent waves effectively relieve constipation in patients with IBS-C (42). Therefore, in clinical practice, practitioners should select different pulse combinations based on the specific IBS subtype and symptoms. Warm acupuncture is a therapeutic method that combines acupuncture and moxibustion. This technique involves threading a moxa roll onto the needle shaft or rubbing a small amount of moxa wool onto the needle tip and igniting it after achieving “deqi” during needling. This therapy is also used to treat IBS (43, 44). Relevant clinical studies show that warm acupuncture can effectively alleviate anxiety and depression in IBS patients (45). Relevant studies have confirmed that acupoints such as Tianshu (ST25), Zusanli (ST36), and Shangjuxu (ST37) are commonly used in warm acupuncture therapy for treating gastrointestinal disorders (33, 46). These acupoints belong to the Stomach Meridian of the Foot-Yangming, which is effective in treating gastrointestinal disorders. Warm acupuncture therapy uses thermal stimulation to continuously activate the meridian's qi, thereby alleviating symptoms associated with IBS.

Previously, in animal studies, most of the outcome measures used to evaluate the effectiveness of acupuncture in alleviating depressive symptoms associated with IBS involved measuring changes in relevant hormone levels in rats, such as corticotropin-releasing factor (CRF) and serotonin (5-HT). In future studies, the types of outcome measures used in animal studies could be expanded to enhance experimental accuracy; in clinical studies, most outcome measures rely on the results of relevant questionnaires, which are overly subjective. Future studies may incorporate corresponding objective measures, such as brain MRI and electroencephalography (EEG), to ensure the objectivity of the results. Meanwhile, most acupuncture treatments for IBS focus on simple acupuncture, heat acupuncture, and electroacupuncture; future research could prioritize both clinical studies and animal experiments to explore different acupuncture methods, thereby providing new therapeutic insights for clinical practice and guiding clinical application.

#### 4.3.2 Acupoint thread implantation

As the number of patients with IBS increases year by year, acupoint thread implantation has been adopted as a clinical treatment modality and has demonstrated good therapeutic efficacy (47–49). Acupoint thread implantation is a therapeutic method that involves embedding medical catgut or protein thread into specific acupoints; this approach achieves therapeutic goals—such as stimulating meridian energy, harmonizing qi and blood, and preventing disease—by providing continuous stimulation to the acupoints (50, 51). Modern research shows that the primary symptoms of IBS are recurrent abdominal pain and bloating, and acupoint thread implantation therapy can effectively alleviate these symptoms in IBS patients (52, 53). Relevant meta-analyses confirm that acupoint thread implantation therapy can effectively improve treatment efficacy and reduce the recurrence rate in patients with IBS-D (54). Related studies confirm that acupoints such as Zhongwan, Guanyuan, and Shangjuxu are widely used in the clinical treatment of IBS and have achieved good therapeutic results (55, 56). Since the aforementioned acupoints are mostly located in areas with thick muscle tissue, acupoint thread implantation therapy can enhance stimulation at these points, thereby providing sustained relief of symptoms in IBS patients and prolonging the therapeutic effect. At the same time, it is worth noting that practitioners must adhere to aseptic techniques during clinical procedures. Furthermore, when treating different acupoints, practitioners must pay attention to the depth and angle of needle insertion to avoid causing pain or other adverse reactions to the patient during the procedure. Currently, there are few multicenter, large-sample, randomized controlled trials; in the future, more such studies could be conducted to identify best practices and provide a basis for clinical application. In addition, future research could focus on animal studies of this method for treating IBS to provide evidence for the underlying mechanisms supporting its clinical use.

#### 4.3.3 Associated symptoms of IBS

According to CiteSpace visualization analysis, changes in bowel habits and abdominal pain caused by chronic visceral hyperalgesia are common accompanying symptoms of IBS in clinical practice. It is worth noting that abdominal pain is a symptom present in all subtypes of IBS, and visceral hyperalgesia is the primary mechanism underlying abdominal pain in IBS patients (57, 58). According to relevant data analysis, different subtypes of IBS exhibit distinct pain patterns: prolonged duration, widespread distribution, and more severe abdominal pain—which may even radiate to the back—are characteristic of IBS-C, whereas abdominal pain associated with bowel movement frequency and consistency is characteristic of IBS-D (59). Clinical studies show that increased *β*-adrenergic activity can lead to visceral hypersensitivity in IBS patients. Furthermore, a comparison of clinical studies and animal experiments revealed that increased 5-HT2B receptor expression can induce TRPV1 expression; the expression of this receptor can induce pain hypersensitivity in IBS-D patients, leading to abdominal pain. This research provides new insights and approaches for the clinical treatment of IBS (60, 61). Relevant experiments have confirmed that acupuncture can alleviate abdominal pain by inhibiting visceral hypersensitivity through neuromodulation of the peripheral and central nervous systems (62–64). Relevant animal studies have shown that acupuncture can alleviate abdominal pain symptoms in IBS rats by reducing extraintestinal hypersensitivity, regulating P2X4 expression, and modulating spinal cord microglia (65). Currently, relevant research has confirmed that acupuncture can suppress abnormal bile acid metabolism and farnesoid X receptor (FXR) expression to alleviate visceral hypersensitivity (66); this study lays a theoretical foundation for future research. Relevant animal studies have shown that moxibustion can reduce visceral sensitivity in rats with IBS by downregulating signaling pathways such as SCF/c-kit, thereby alleviating abdominal pain symptoms (67, 68). Moxibustion activates the spinal motor opioid and orphanin-fq systems, reduces levels of corticotropin-releasing hormone (CRH) in the hypothalamus, and decreases norepinephrine-1 and norepinephrine receptor-1 levels, thereby alleviating visceral hyperalgesia (33, 69, 70).

Studies show that changes in bowel habits are a primary symptom of IBS, with diarrhea being the main symptom of IBS-D and functional constipation being the main symptom of IBS-C (71, 72). Early studies found that diarrhea symptoms in IBS patients are associated with small intestinal permeability (73, 74). Currently, relevant experiments have confirmed that impaired active ion secretion and absorption, an imbalance between neurotoxic and protective metabolites, and polymorphic expression of serotonin signaling are the causes of changes in bowel habits in IBS (75–77). Clinical studies have confirmed that acupuncture can effectively alleviate diarrhea and constipation symptoms in IBS patients (78, 79). Furthermore, relevant studies indicate that external TCM therapies—such as auricular acupuncture, tuina massage, and acupoint plaster application—can effectively alleviate diarrhea or constipation symptoms in IBS patients (13, 80, 81). Although current research has confirmed that external TCM therapies are effective in relieving clinical symptoms of IBS, their underlying mechanisms of action remain unclear. Currently, the relief of abdominal pain is primarily assessed using the Visual Analog Scale (VAS), but this method yields results that are not entirely objective. Therefore, future research could combine this approach with modern technologies—such as ultrasound and muscle tone measurement—to establish appropriate outcome measures to guide clinical practice. Furthermore, observations of changes in bowel habits among IBS patients are primarily based on records kept by the patients or their families. In the future, these observations could be combined with current artificial intelligence technologies to enable intelligent monitoring; this approach would reduce the burden on patients while improving the accuracy and reliability of the assessments.

### 4.4 New trends

According to a CITESpace network analysis, current research focuses primarily on the mechanisms of action of traditional Chinese medicine (TCM) external therapies for treating IBS, the principles of acupoint selection, and moxibustion therapy.

#### 4.4.1 Mechanism studies

Currently, the pathogenesis of IBS remains unclear (72, 82). A comprehensive analysis of modern research suggests that the brain-gut interaction is the core mechanism in the treatment of IBS (83, 84), with immune regulation (85–89), gut microbiota regulation (90–92), and neural regulation pathways (93, 94) being the primary influencing factors in this mechanism. Among the external treatment methods in Traditional Chinese Medicine (TCM) used to regulate the immune system in IBS, multiple studies have shown that electroacupuncture can treat IBS by protecting the intestinal mucosal barrier through the stimulation of immune pathways such as mast cells, tryptase, PAR-2, and myosin light chain kinase (37, 95, 96). A clinical trial concluded that moxibustion can treat symptoms associated with this disease by correcting abnormal immune dysfunction (23, 72). Furthermore, regarding this therapy's ability to improve the gut microbiota in IBS, relevant animal studies have shown that acupuncture can alleviate diarrhea symptoms in IBS patients by increasing gut microbiota such as *Citrobacter* and *Bacteroides* (97, 98). It is worth noting that external treatments in Traditional Chinese Medicine can effectively improve IBS-related symptoms by regulating neurotransmitters. Multiple animal studies have confirmed that this therapy can alleviate intestinal dysfunction in rats by modulating the brain-gut axis through the regulation of neuroactive substances such as 5-HT and *β*-hydroxybutyrate (99, 100). Furthermore, relevant clinical trials have found that this therapy can improve patients' intestinal microenvironment by modulating the vagus nerve–brain–gut axis (80, 101). Therefore, researchers in this field should intensify studies on the underlying mechanisms to provide better treatment strategies for clinical practice. At present, no comprehensive framework has been established regarding the mechanisms underlying the use of external TCM therapies for treating IBS. In the future, researchers should strengthen collaboration to integrate various mechanistic studies and develop a more comprehensive framework that can better elucidate the biological mechanisms of external TCM therapies for treating IBS.

#### 4.4.2 Principles of acupoint selection

Current research focuses primarily on the selection of acupoints and the investigation of their mechanisms of action. Although acupuncture is widely used in clinical practice as an effective treatment for alleviating IBS symptoms (102), data analysis indicates that acupoints such as Tianshu (ST25), Shenque (CV8), Zhongwan (CV12), and Zusanli (ST36) are currently the main points used to treat IBS. Among these, Tianshu (ST25) is the most frequently used acupoint in clinical practice (78, 103). Clinical trials have confirmed that electroacupuncture at the Tianshu (ST25) acupoint reduces glucose metabolism in brain regions such as the left cingulate gyrus and right insula, and that this therapy can alleviate abdominal pain and diarrhea symptoms in IBS patients (104). An animal study on moxibustion confirmed that applying moxibustion to the Tianshu (ST25) acupoint reduces the expression of prokinin-1 and prokinin receptor-1 in hyperalgesic rats; this therapy can effectively relieve abdominal pain in patients with IBS (70, 105, 106). Relevant clinical studies show that the Ba Liao acupoints can alleviate visceral hypersensitivity by regulating the gut microbiota, inhibiting mast cells, and blocking TRPV1 neurons, thereby relieving abdominal pain symptoms in patients with IBS (107). Clinicians use the Tianshu acupoint in combination with Zusanli, Zhongwan, and other acupoints to treat IBS, and this method effectively relieves abdominal pain symptoms in patients with IBS. Currently, clinical research on acupoints remains inconsistent. Future efforts should focus on data analysis and clinical trials regarding acupoint selection to identify the acupoints that yield the best therapeutic outcomes and to investigate the effects of different acupoints on the various syndromes exhibited by patients with IBS. This will enable clinicians to develop personalized treatment plans for individual patients and improve treatment outcomes. At the same time, more animal studies should be conducted to further explore the mechanisms underlying acupoint therapy and provide guidance on the biological mechanisms for clinical application.

#### 4.4.3 Moxibustion

Moxibustion is a therapeutic method that involves burning herbal preparations containing combustible substances on acupoints or specific areas of the body; this therapy uses the resulting thermal stimulation to treat chronic conditions such as arthritis and digestive disorders (108, 109). Artemisia is the primary combustible material used in moxibustion, and the thermal stimulation from moxibustion helps balance yin and yang, regulate the internal organs, and promote overall health (110). Relevant studies have confirmed that moxibustion can treat IBS by regulating amino acid metabolism, brain signaling, energy metabolism, and anti-inflammatory pathways (102, 111). Clinical studies have confirmed that moxibustion can effectively alleviate symptoms such as abdominal pain and diarrhea in IBS patients; at the same time, this therapy is highly safe and suitable for clinical application (112). Relevant meta-analyses indicate that moxibustion plays an important role in the treatment of diarrhea-predominant IBS (108, 110). Animal studies have suggested that ginger-separated moxibustion, mild moxibustion, or laser moxibustion can all activate TRPV1 channels, thereby inhibiting the pathological activation of TRPV1 ion channels in the colon and alleviating abdominal pain. Furthermore, the different thermal stimuli produced by these three moxibustion methods on specific sites can yield varying therapeutic effects on the disease (113). Therefore, in clinical practice, dynamic and repeated thermal stimulation should be employed when using moxibustion therapy to treat IBS in order to enhance treatment efficacy. A clinical study investigating the treatment of IBS with moxibustion at different temperatures concluded that maintaining mild moxibustion at 43°was more effective than at 37° in relieving abdominal pain symptoms, altering stool consistency, and improving quality of life in patients with IBS-D (114). Therefore, precise temperature control is crucial in the clinical application of moxibustion therapy. Future researchers may focus on studies specific to the duration of moxibustion, the distance at which it is applied, and the treatment of patients with different diseases and symptoms, so that clinicians can tailor treatment plans to individual patients and provide more targeted care.

## 5 Strengths and limitations

The use of CiteSpace for visual and analytical synthesis of the literature is a significant strength of this study. Discussion of the visualization results helps reveal current research hotspots and future trends in this field, guide future research, and provide a reference for researchers, thereby laying a stronger foundation for future studies. However, this study also has limitations: This study selected only literature from the China National Knowledge Infrastructure (CNKI) database, the Web of Science database, and the PubMed database; other databases, such as Embase and the Cochrane Library, were not included. Furthermore, this study encompasses relevant research published to date, and newly published articles in this field will not be included in this study. Therefore, to address this issue, researchers must update and summarize their new research or projects. Furthermore, this study includes literature in Chinese and English but does not cover research in other languages; at the same time, the literature reviewed in this study is limited to review articles and research articles. Nevertheless, the bibliometric methods employed in this study effectively summarize the current state of the field and provide new insights into research hotspots and trends.

## 6 Conclusion

This study comprehensively and objectively analyzed the relevant literature on the treatment of IBS using external therapies in Traditional Chinese Medicine from 1990 to 2026, generating visual maps of annual publication volumes, authors, and keywords. Current research indicates that the field holds great promise for future development, but collaboration among institutions is also crucial. Research hotspots in this field primarily focus on acupuncture, acupoint thread implantation therapy, and IBS-related comorbid symptoms. The most common comorbid symptoms observed clinically are changes in bowel habits and abdominal pain caused by visceral hyperalgesia. Therefore, clinicians should prioritize alleviating these symptoms to improve patients' quality of life. Research trends are primarily focused on the mechanisms underlying the treatment of IBS with external TCM therapies, the principles of acupoint selection in TCM, and moxibustion therapy. Therefore, in clinical practice, physicians should administer treatments guided by relevant theories to improve treatment efficacy. At the same time, this study suggests that these therapies can not only effectively alleviate patients' clinical symptoms but also alleviate their accompanying symptoms.

## Data Availability

The original contributions presented in the study are included in the article/supplementary material, further inquiries can be directed to the corresponding author/s.
